# Health management and pattern analysis of daily living activities of people with dementia using in-home sensors and machine learning techniques

**DOI:** 10.1371/journal.pone.0195605

**Published:** 2018-05-03

**Authors:** Shirin Enshaeifar, Ahmed Zoha, Andreas Markides, Severin Skillman, Sahr Thomas Acton, Tarek Elsaleh, Masoud Hassanpour, Alireza Ahrabian, Mark Kenny, Stuart Klein, Helen Rostill, Ramin Nilforooshan, Payam Barnaghi

**Affiliations:** 1 Department of Electrical and Electronic Engineering, University of Surrey, Surrey, United Kingdom; 2 Surrey and Borders Partnership NHS Foundation Trust, Leatherhead, Surrey, United Kingdom; University of Illinois at Urbana-Champaign, UNITED STATES

## Abstract

The number of people diagnosed with dementia is expected to rise in the coming years. Given that there is currently no definite cure for dementia and the cost of care for this condition soars dramatically, slowing the decline and maintaining independent living are important goals for supporting people with dementia. This paper discusses a study that is called Technology Integrated Health Management (TIHM). TIHM is a technology assisted monitoring system that uses Internet of Things (IoT) enabled solutions for continuous monitoring of people with dementia in their own homes. We have developed machine learning algorithms to analyse the correlation between environmental data collected by IoT technologies in TIHM in order to monitor and facilitate the physical well-being of people with dementia. The algorithms are developed with different temporal granularity to process the data for long-term and short-term analysis. We extract higher-level activity patterns which are then used to detect any change in patients’ routines. We have also developed a hierarchical information fusion approach for detecting agitation, irritability and aggression. We have conducted evaluations using sensory data collected from homes of people with dementia. The proposed techniques are able to recognise agitation and unusual patterns with an accuracy of up to 80%.

## Introduction

Dementia is a syndrome consisting of a group of symptoms that suggest progressive brain dysfunction and global impairment of cognition or intellects. Dementia usually manifests as problems with memory and orientation, thinking processes, learning and language, calculation and comprehension, judgement and emotion and several other symptoms [1, 2] to an extent that it decreases functionality in Activities of Daily Living (ADL) [3]. There are currently around 46.8 million people living with dementia around the world, with numbers set to increase to 74.7 million by 2030 and 131.5 million by 2050 [4], and it has a direct impact on an individual’s health, quality of life, and ability to live independently. World Alzheimer’s Report 2015 reveals that the annual societal and economic cost of dementia is 818 billion USD, and is expected to increase to trillion dollar disease in the next few years. The reports show that the cost of dementia has increased by 35% since 2010. Given that there is no current definite cure/treatment for dementia, slowing the decline, stabilisation, and maintaining independent functioning are important goals in supporting people with dementia [5]. One approach to enhance independent living is to utilise assistive technologies in people’s homes. To date, several studies have evaluated the benefits of assistive technologies in the elderly demographic [6–10]; however, very few have focused on individuals with dementia [3, 11, 12].

Traditionally, monitoring the functional capability and cognition of people with dementia is assessed through questionnaires or clinical interviews during clinic visits. However, recent technology-based assessment systems can facilitate continuous monitoring of patients in their own living environments. This provides continuous and fine-grained information about the individuals’ health and well-being (e.g. their daily activity patterns, physical ability and state) that cannot be obtained through infrequent and intermittent visits. Analysing the continuously monitored data provides reliable long-term assessments and can generate actionable information about an individual’s activities, trends and routines. To date, several studies have focused on the activity recognition and routine detection using in-home sensors [10, 13–18]. For instance, [10, 13] used passive infrared (PIR) motion sensors to define activities for a particular location, [14] used PIR motion densities to capture the activity level, and [15, 16] analysed the sequential pattern of activities recorded via PIR sensors to investigate changes in health conditions. Furthermore, some studies have focused on the detection of cognitive changes using a combination of motion, pressure, and door sensors [10, 17, 18]. Generally, activities are detected either through specific rules or by applying learning techniques using labelled data—which is not available for uncontrolled real-world environments. Therefore, in this work, we aim to provide a robust and more generic approach to discover the underlying pattern of participants’ activities given a large data set that contains unlabelled data (see Section ‘Macro assessment’). In addition, most of the existing research focus exclusively on physiological parameters or environmental data, and often, neglect their correlation. However, this study integrates physiological and environmental data and utilises adaptive algorithms where the relationship between environmental and physiological data is considered in extracting health and well-being related information (see Section ‘Micro assessment’).

Applying data analytics and machine learning algorithms has a great potential for a preventative approach in healthcare services. Technology-based monitoring systems provide an opportunity to support patients with chronic conditions, who live in their own homes [3]. Several studies have demonstrated that such systems enhance quality of life, prolong independent living and reduce caregivers’ burden and healthcare costs in general, while maintaining the patient’s safety [7, 8, 11]. The goal of the current work is to develop an innovative living environment which empowers people with dementia and their caregivers to benefit from healthcare support and have better quality of life in their own homes. We have developed an Internet of Things (IoT) platform for a technology integrated health management (TIHM) system which facilitates automated monitoring and assists healthcare practitioners to support people with dementia and their caregivers. In TIHM, we have developed machine learning and data analytics algorithms that combine physiological and environmental data to learn and discover changes in patients’ health and well-being. This allows early identification of patients’ needs and provides better quality of care for people with dementia [19].

**Fig 1:**
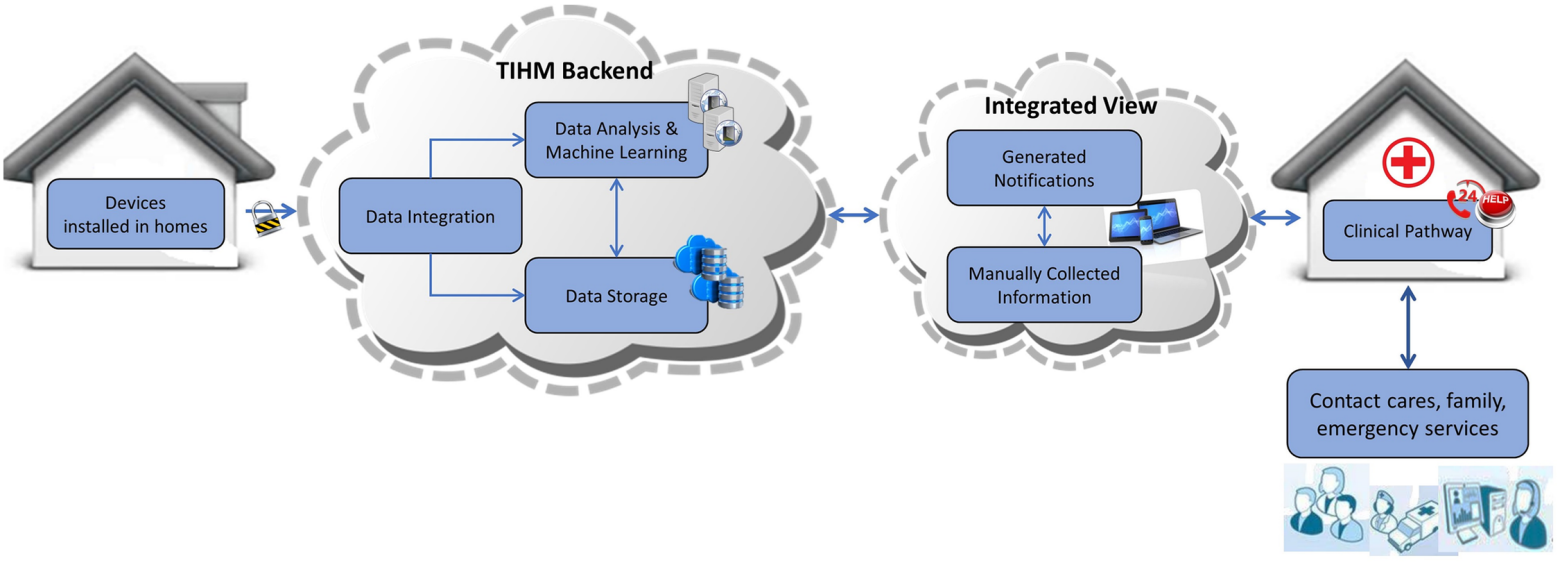
The high-level overview of the interactions in TIHM.

A higher-level overview of the interactions in the TIHM architecture is shown in Fig 1. The system has four components: 1) sensors installed in homes, 2) TIHM back-end system including the integration tool, storage and analysis methods, 3) the user interface for data visualisation and presenting clinical, environmental and technical alerts as well as learned insights and actionable information and 4) clinical pathways where a group of healthcare practitioners monitor the data 24/7 and interact with the patients, caregivers and respond to their healthcare needs around the clock.

## Problem statement

In this paper, we describe machine learning algorithms that aim to support the physical well-being of people with dementia by analysing raw observations and measurement data alongside environmental data, collected from their homes as shown in Fig 1. The collected data in the TIHM project is integrated and processed using several data analytics and machine learning algorithms to generate personalised notifications based on patients’ needs, aligned to the parameters set by the clinical team. This information is monitored around the clock by a group of healthcare practitioners (i.e. the monitoring team) who take appropriate decisions by following a clinical algorithm taking into account the collected data and generated notifications (i.e., actionable information). This approach allows the clinical team to provide more effective support at the earliest point of need to prevent escalating situations. At the same time, the monitoring team validates the generated notifications and this information is used for the evaluation of the developed machine learning techniques. These algorithms include (1) rule-based reasoning methods for vital measurements and providing adaptive learning mechanisms to make them more personalised and (2) machine-learning techniques to learn the individuals’ activities/patterns and detect unusual deviations.

In this study, we propose pattern detection algorithms that provide a holistic view of patients’ activity and routines. Analysing patients’ data at different temporal granularity has enabled us to develop automated procedures for macro and micro assessments. The objective of our proposed macro assessment is to look for unusual patterns on a larger time span (i.e., daily or weekly scale). On the other hand, the micro assessment framework is more focused on detecting underlying activity patterns that can be used to infer the day-to-day well-being of people with dementia. In our study, we have investigated the detection of patterns that directly correlate with Agitation, Irritation and Aggression (AIA) which are commonly observed in people with dementia.

## Materials and methods

The study protocol for this work has been reviewed and approved by the South East Coast Surrey NHS Research Ethics Committee. We obtained informed consent from all the study participants. Each participant was assessed according to Mental Capacity Act guidelines (available at https://www.gov.uk/government/publications/mental-capacity-act-code-of-practice) by a fully qualified researcher who has completed a mandatory clinical practice course. Participants understood the study and were able to understand the consent process.

Participants in this study have had a confirmed diagnosis of dementia (mild to moderate) within, at least, the past three months and have been stable on dementia medication. Participants live in Surrey area and have been receiving a minimum of 10 hours care per week from their family or a formal caregiver. Participants were recruited from the dementia research list held by the research and development (R&D) team in Surrey and Borders Partnership NHS Foundation Trust (SABP), self-referral and referral from general practitioners (GPs). Each participant was recruited over a six months trial period. During the trial, the environmental data was continuously recorded via sensors installed in participants’ homes. The environmental sensors consist of 2 PIR sensors, 4 motion sensors, 2 pressure sensors, 1 door sensor and 1 central energy consumption monitoring device. The physiological data was recorded twice a day by participants using the Bluetooth-enabled medical devices. The physiological data includes blood pressure, heart rate, body temperature, weight and hydration readings.

All the methods were performed in accordance with the ethics guidelines and regulations. The proposed macro and micro assessment methods are described in the following sections.

### Pattern analysis: Macro assessment

The existing works and dementia trials have shown the high variability of daily activities in people with dementia which leads to dissolving patterns in their daily routines. The existing studies demonstrates that individuals with dementia exhibit one or more symptoms which may occur at any time of the day; meaning that people with dementia have more non-structured routine compared to healthy individuals [3, 20]. According to [3], trend analysis can help identify behavioural anomalies in people with dementia that aid clinicians’ decision making in providing health and care support.

Given the above, analysing the daily routine plays an important role in monitoring the health and well-being of people with dementia. We have developed algorithms which provide long-term information about the participants’ routines by analysing the sequence of their daily activities (inferred from sensory observations and measurements). This information is used to observe if participant is constantly deviating from her/his routine, i.e. exhibiting unusual patterns. In this case, further investigation is required to distinguish whether these deviations are related to the technical infrastructure faults, environmental changes (i.e., having visitors) or are associated with possible condition change which may indicate the progression of the cognitive decline in people with dementia.

In this section, unusual patterns are referred to as ‘anomaly’ which is defined based on the sequence of activities; i.e. sequence alignment is used to detect unusual pattern in daily activities. To analyse the daily routines and detect unusual patterns, a ‘Markov chain’ is employed to model the activity sequences and an ‘entropy rate’ has been calculated to quantify the regularity of an individual’s patterns in their day-to-day life. These concepts are discussed below.

#### Markov chain

**Fig 2:**
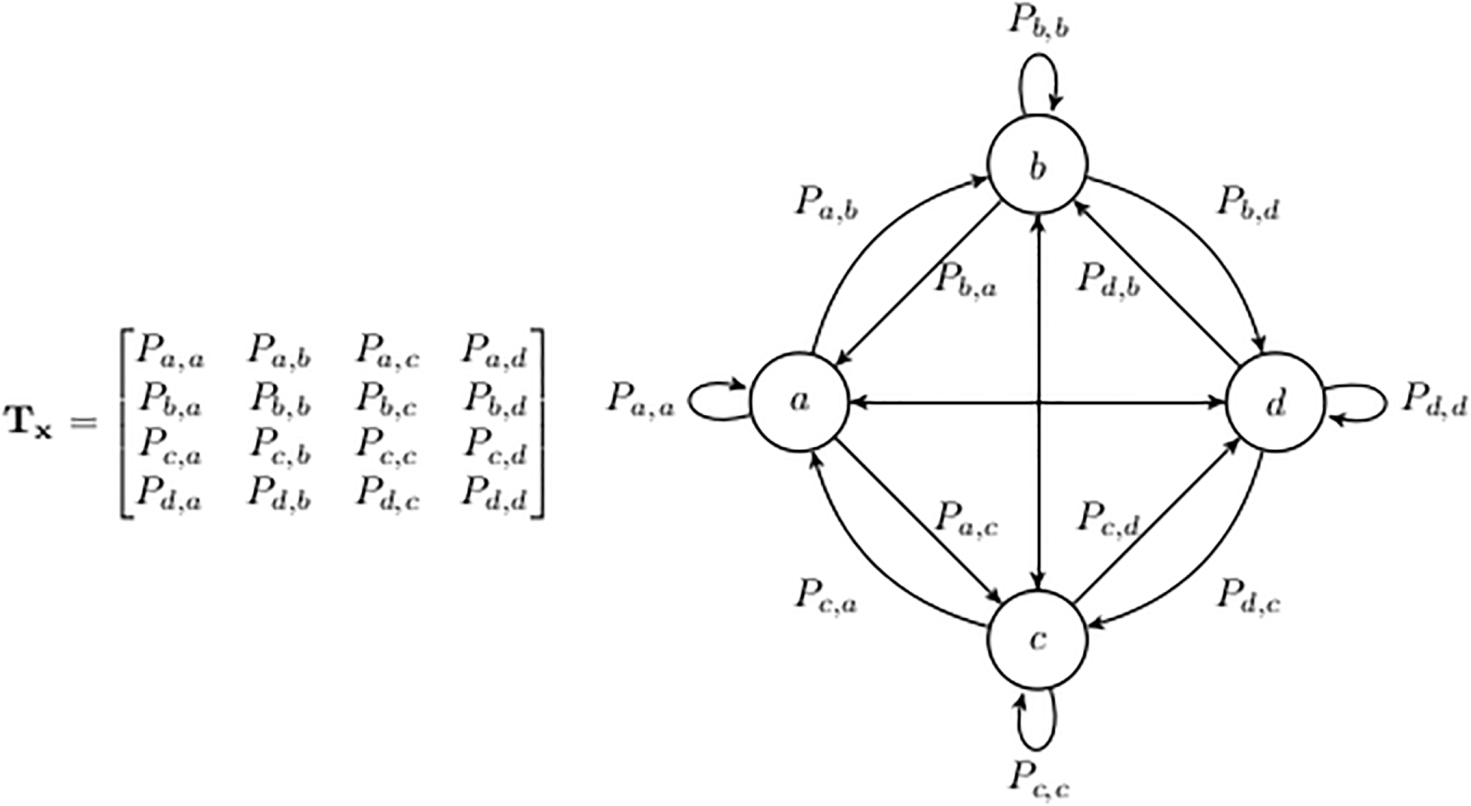
A sample Markov model with 4 states.

Since anomaly is defined based on the sequence of activities, it is necessary to profile an individual’s daily routine using a probability-based model where the probability of each step (or activity) depends on the previous step [21]. The simplest such model is a conventional Markov chain in which a stochastic process **x** = {*x*_1_, *x*_2_, ⋯, *x*_*l*_} consists of a finite number of states **S** = {*s*_*a*_, *s*_*b*_, ⋯, *s*_*f*_} (where *x*_*i*_ ∈ **S**) and the probability of each step *x*_*i*_ only depends on the value of the preceding step *x*_*i*−1_ and not on the entire sequence [21–23], i.e.

$$\begin{array}{c} P\left(x_{i}|x_{i-1},\cdots ,x_{1}\right)=P\left(x_{i}|x_{i-1}\right)=p_{i-1,i} \end{array}$$ (1)

in which *P*_*i*−1,*i*_ is the transition probability of *x*_*i*−1_ → *x*_*i*_. Each Markov chain can be represented as a Markov model with a corresponding transition matrix. For instance, in Fig 2, a Markov chain **x** with four states (*a*, *b*, *c*, *d*) is represented with the transition matrix **T**_**x**_.

#### Entropy rate

Generally, the day-to-day activities in home can be seen to be random or regular in short or long period of time, as daily activities do not necessarily follow a specific pattern in short time spans but over long-term observations some specific patterns could be observed. It can be assumed that each person has a ‘ground-state’ of typical routines which are mixed with some fluctuations. We use an information theory based parameter (i.e., entropy) to evaluate these fluctuations [24].

Entropy is a well-known measure for quantifying information and indicating the degree of random occurrence in a succession (in our case the Markov chain) and it has been extensively studied for pattern detection [23, 24]. Given the transition matrix **T**_**x**_, the entropy value is calculated as [22, 25, 26]:

$$\begin{array}{c} \epsilon =-\sum_{\alpha \beta }P_{\alpha \beta }.log\left(P_{\alpha \beta }\right) \end{array}$$ (2)

in which *ϵ* is the entropy value; *P*_*αβ*_ are the transition probabilities for a transition from state *α* to state *β* in a Markov chain. Larger *ϵ* values indicate higher uncertainty level (randomness) and the entropy value reaches its maximum if all the states occur with equal probabilities. The entropy defined in Eq (2) only depends on the ‘transition probabilities’ between states and it ignores the state probabilities. To incorporate the state probabilities, we use the entropy rate value which is defined as [27, 28]:

$$\begin{array}{c} \xi =-\sum_{\alpha \beta }P_{\alpha }P_{\alpha \beta }.log\left(P_{\alpha \beta }\right) \end{array}$$ (3)

in which *ξ* is the entropy rate. The entropy rate depends on both the state probability (*P*_*α*_) and the transition probability (*P*_*αβ*_) which enhances the discrimination ability of the algorithm. For instance, if *P*_*αβ*_ = *P*_*βα*_ and *P*_*α*_ ≠ *P*_*β*_, the calculated entropy rate differentiates between transition patterns from *α* to *β* and *β* to *α*, while the entropy value assigns the same value to both patterns.

#### The proposed algorithm

We have used a Markov chain model and entropy rate to develop an algorithm that identifies the daily routines and detects the unusual patterns of people with dementia. We first construct a Markov chain model based on an individual’s historical data to identify their regular daily routine or sequence of events. Although an individual’s activities can be quite complicated, they usually follow a sequence of events in specific locations and time ranges within each day [24]. In other words, the proposed algorithm observes the participants’ day-to-day activities in a quantitative fashion and detects anomalies by analysing the deviation and differences in the distribution of the Markov model. The entropy rate of the historical data is used to define the ‘deviation boundaries’ and any test data exceeding these boundaries is detected as an anomaly. In order to select the deviation boundaries (i.e. defining how much deviation is permitted), the historical data is divided into two sets as ‘training and verification’. The training set is used to construct the Markov model by learning the state transition probabilities while the verification set is used to define a confidence threshold for the deviation range.

#### Data pre-processing

The environmental data used in this study is collected from a subset of passive sensors installed in different locations within residential homes. We have deployed 9 sensors in total: 2 PIR sensors (installed in the hallway and living room), 3 motion sensors (one in the kitchen and 2 on the bedroom and bathroom doors), 2 pressure sensors (installed on the chair and the bed), 1 main entrance door sensor and 1 central energy consumption monitoring device. The data is therefore collected from multiple sources (provided by 7 different companies) in uncontrolled real-world environments, i.e. residential homes. This means that data may be noisy and incomplete (containing missing values) with various sampling rates for different source. We first apply pre-processing techniques to only include those participants who have at least three sensors reporting continuously for the last three months. We then generate the training and verification sets for each of these participants. The raw data is filtered and aggregated over 10-minute intervals; i.e. each day is represented by a 24 × 6 window of 10-minute intervals and each window is an array of *n* elements where *n* is the number of active nodes for one participant. In addition, to perform feature scaling, the aggregated data is normalised such that the activity level of each sensor is ranged between 0 to 10. This ensures that all sensors are represented within the same range, regardless of their sampling rates and type of measurements, where they are equally taken into consideration for further analysis, such as K-means algorithm [29].

Prior to constructing the trained Markov model, the integrated data is clustered into two categories using a K-means algorithm [29]. This is particularly important as individuals typically exhibit different patterns of daily activities on high-active days (e.g. being home most of the day) compared to low-active days. These clusters are sorted based on their total number of observations, so that categories D1 and D2 represent low-active and high-active days, respectively. In the next step, a second K-means algorithm (with *n* centroids) is used to map each *n*-dimensional window to a single state. These states are then used to construct the Markov Model. The transition probabilities between the states are subsequently learned using the training set observations. The learned model is then used to infer an individual’s routine and detect anomalies, as described below. Notice that clusters are estimated only based on the training dataset and the same model has been then re-used for the verification and test sets, meaning that centroids of the K-means clusters have been fixed throughout the analysis.

#### Anomaly detection

To analyse the level of variation/randomness for each participant, the pre-processed dataset, including *D*1 and *D*2 categories, is divided into the training and verification sets. The training sets are used to construct two Markov models with transition matrices **T**_*D*1_ and **T**_*D*2_. This results in two separate models for high activity and low activity days. The number of clusters and models representing them can increase based on a need for more detailed and specific models. In this paper, we demonstrate our proposed solution based on 2 clusters.

In the next step, we quantify the information and analyse the randomness based on the consecutive state transitions. To achieve this, the transition probabilities for the Markov chain are learned and the entropy rate is calculated based on the chain. For instance, the transition probabilities for a Markov chain {*a*, *b*, *a*, *a*, *c*, *b*} are represented with {*P*_*aa*_, *P*_*ab*_, *P*_*ba*_, *P*_*aa*_, *P*_*ac*_, *P*_*cb*_} and the entropy rate is calculated as

$$\begin{array}{c} \xi =-\sum (\;P_{a}.P_{aa}.log\left(P_{aa}\right)+P_{a}.P_{ab}.log\left(P_{ab}\right)+\cdots +P_{c}.P_{cb}.log\left(P_{cb}\right)\;) \end{array}$$ (4)

where *P*_*aa*_ represents the state probability and *P*_*ab*_ represents the state transition probability. After calculating the entropy rate for the training data (*ξ*_*T*_), this process is repeated to calculate an entropy rate for each day from the verification set, i.e. generating ξ_*V*_ = {*ξ*_1_, ⋯, *ξ*_*v*_} in which *v* is the number of verification days. These values are used to determine the expected deviation boundaries by calculating the confidence interval Δ as:

$$\begin{array}{c} \Delta =\xi_{T}\pm \mu \frac{\sigma }{\sqrt{v}}\;\text{where}\;\sigma =\sqrt{\frac{\sum {\left(\xi_{V}-\xi_{T}\right)}^{2}}{v}} \end{array}$$ (5)

in which *μ* is the confidence coefficient. Given the above, anomalies are identified as days in which entropy rates exceed the expected boundaries (*ξ* > Δ), i.e., days that their sequence distribution highly deviates from the trained Markov model.

The proposed method is summarised in S1 Algorithm (see S1–S3 Algorithms). The proposed macro assessment algorithm identifies unusual patterns over a longer time scale (i.e., daily and weekly analysis). The next section describes the micro assessment which is focused on detecting patterns on a short-time span.

### Pattern analysis: Micro assessment

In addition to detecting unusual activity patterns on a macro level, we have developed algorithms to reason at a finer temporal granularity. It allows us to carry micro level automated detection of AIA based on daily activities. AIA are common conditions in people with dementia, in conjunction with memory and sleep problems [30]. A common challenge faced by doctors and caregivers is the detailed and continuous monitoring of AIA, since it is mostly reported by second-hand accounts that are susceptible to bias. This is mainly because the observer is prone to anxiety and stress that leads to erroneous observations. Subsequently, it is of high importance to take into consideration the context in which the observed symptoms were reported, their frequency and detection of any possible drift over time. The AIA symptoms not only adversely affect the health of the patient but also of their caregivers [31]. In order to address the problem, our proposed framework uses contextual information obtained from various combinations of sensing modalities, allowing it to continuously monitor and detect any unusual pattern in the participants’ daily activities.

**Fig 3:**
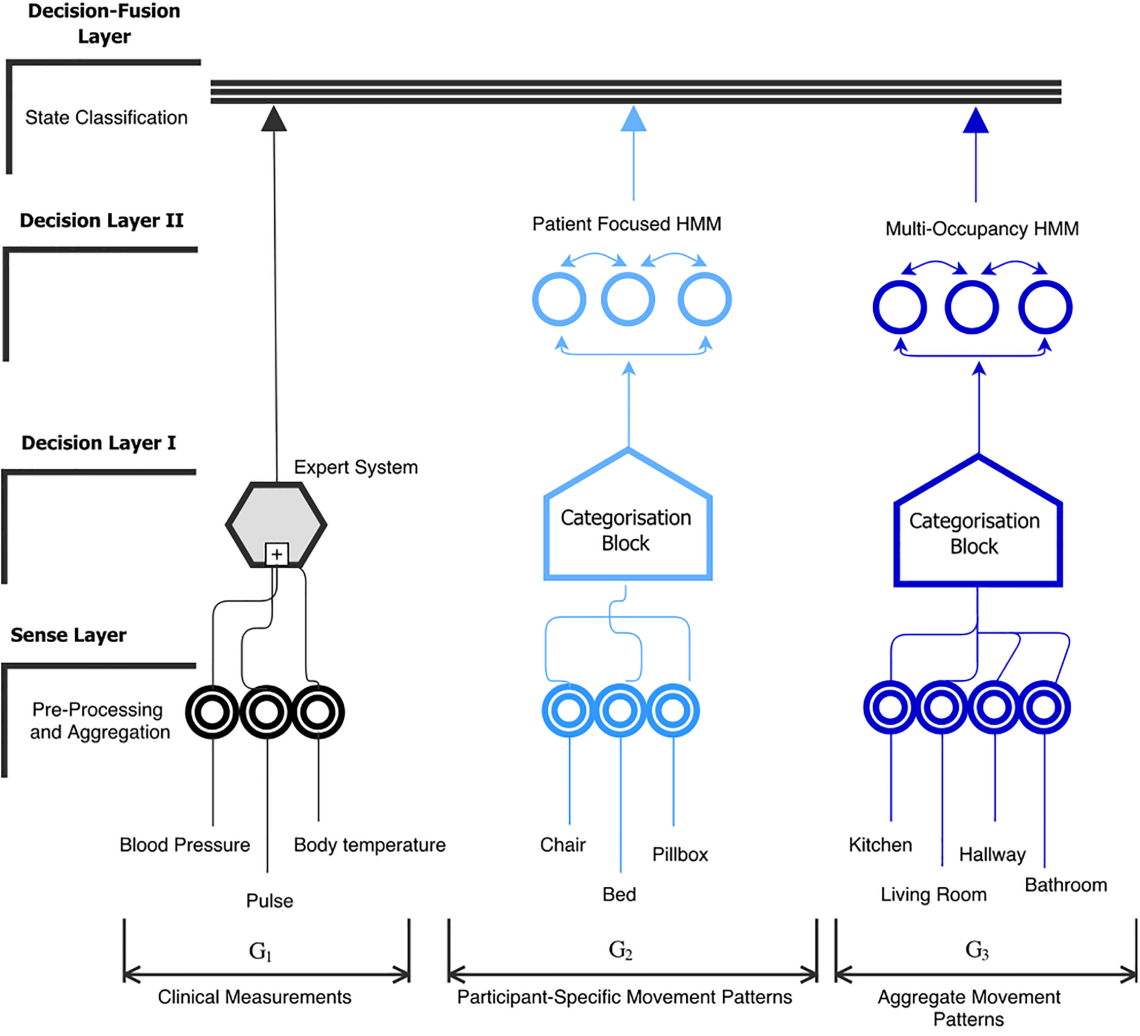
A framework for detecting Agitation, Irritation and Aggression (AIA) in people with dementia.

It is important to note that symptoms of agitation can be imperfectly categorised as “hyperactive” or “hypoactive”. Hyperactive symptoms in dementia include abnormality in the motion pattern, an increased frequency of repetitive actions such as chair movement or leg movement. On the contrary, hypoactive symptoms in dementia exhibit complete disengagement and isolation from activities or depression. We focus on the assessment and detection of hyperactivity in people with dementia and use this to determine the chance of AIA. Our proposed AIA detection algorithm consists of a sensing layer, decision layers and a fusion layer, as shown in Fig 3. The functionality of each of these layers is discussed below:

#### Sensing layer

The sensing layer consists of multiple sensing modalities which are categorised into subgroups *G*_1_, *G*_2_ and *G*_3_ based on the context of the collected data. For example, the physiological monitoring sensors belong to *G*_1_ and are used to collect blood pressure, heart rate and body temperature of the participant. These sensors are wirelessly connected to a gateway, enabling them to automatically relay the measurements to a server upon activation. On the other hand, *G*_2_ and *G*_3_ consists of environmental sensory devices, see Fig 3. As shown, *G*_2_ includes motion activity patterns collected from the chair, pillbox and bed of the participant, whereas *G*_3_ contains the motion data that is collected from sensors deployed in the living room, hallway and kitchen. It can be seen from Fig 3 that the deployment of *G*_2_ sensors are closely associated with the areas of participant’s movement, whereas *G*_3_ sensors record the multi-occupancy movement pattern within a home. The formation of groups *G*_2_ and *G*_3_ not only helps us to contextualise movement activity (i.e., allowing us to decouple participant specific motion activity in comparison to the aggregated movement indicator) but also allows us to learn the relationship between AIA events and specific activity patterns. Each subgroup in the sensing layer pre-processes the collected observations, aggregates it and forwards it to the respective processing modules in decision layer I.

#### Decision layer I & II

The decision layer I receives the measurements from the sensing layer and processes them further using expert system decision scoring and categorisation algorithms as detailed in S2 and S3 Algorithms, respectively (see S1–S3 Algorithms). The expert decision scoring algorithm takes an input from *G*_1_ that consists of physiological symptom monitoring sensors *CS*_*i*_, where *i* ∈ *G*_1_. As shown in S2 Algorithm, the decision score *D*_*CS*_ is calculated based on comparing the observations *O*_*i*_ belonging to *CS*_*i*_ against the standard clinical thresholds $T_{i}^{H}$ and $T_{i}^{L}$ (provided by the clinical experts). For each *CS*_*i*_, a threshold comparison is performed and subsequently the value of *D*_*CS*_ is incremented only if the *O*_*i*_ is outside the threshold values. The decision score is then forwarded to the fusion layer for final inference as shown in Fig 3.

On the other hand, the categorisation algorithm is applied independently to the measurements obtained from *G*_2_ and *G*_3_. At first, a mapping procedure is performed in which hourly aggregated measurements *P*_*x*_ from each of the respective sensors within a group is mapped to a unique alphabetical label. Once the label assignment for each sensor is complete, the algorithm outputs a string (*S*) by assigning the generated labels as detailed in S3 Algorithm. The label assignment step is performed as follows. Firstly, a label set *A*_*x*_ corresponding to each sensor within a target group is pre-stored in the database. *A*_*x*_ consists of labels that represent two states: normal and abnormal. Each label is unique such that intersection amongst any label set would result in an empty set. The mapping of *P*_*x*_ to a specific label from *A*_*x*_ is determined by threshold comparison. This requires estimation of appropriate upper and lower threshold boundaries $B_{x}^{u}$ and $B_{x}^{l}$ for each target sensor within *G*_2_ and *G*_3_.

To estimate the threshold values for label assignment, we employed an adjusted box-plot method as proposed by Hubert and Vandervieren [32]. We used three months of historical motion sensor data for each target participant as an input to the box-plot method. A standard box-plot gives information regarding the shape, variability, and centre (or median) of a data set and is often used to detect outliers. However, an advantage of using adjusted box-plot in comparison to box-plot is the ability to handle skewed data distributions. Since our data is noisy, we selected the adjusted box-plot method to compute the threshold values of $B_{x}^{u}$ and $B_{x}^{l}$. The label assignment makes use of these boundary values to select a label from *A*_*x*_. For example, if the observed measurement falls within the boundary values, a label corresponding to a normal state would be selected or vice-versa. Once the mapping is finished the categorisation block of *G*_2_ and *G*_3_ generates the sequence of labels *S*_*G*_2__ and *S*_*G*_3__, respectively, and forwards it to the decision layer II for further processing.

The label sequences *S*_*G*_2__ and *S*_*G*_3__ are passed on to their respective Hidden Markov Models (HMM) as shown in Fig 3. HMM [33] is well known to model sequential data and it addresses the problem of finding hidden states that might have generated the observations. HMM is based on stochastic processes and computes the joint probability of a collection of hidden states and observed variables. In our case, we are interested to find if the observed movement patterns exhibit participants’ normal routine or symptoms of agitation based on the hourly sequences *S*_*G*_2__ and *S*_*G*_3__ fed in by decision layer I. To be able to identify anomalies in the participant’s specific movement pattern as well as the multi-occupancy movement pattern, we have developed independent HMM models for *G*_2_ and *G*_3_. A person can stay in one state for several hours and then potentially change to a different state. The HMM works on the principle that a transition matrix describing the probabilities of transitioning between states can be learned from the observation sequence and that prior probabilities of starting in a particular state are known, so that the probability of sequence of states can be computed. In this work, participant-specific and multi-occupancy HMM models learn the joint probability of observing a sequence of labels and the hidden state of the participant (i.e., AIA or normal).

Our HMM model can be characterised by λ = {**T**, **B**, *π*}, where **T** is a transition matrix representing the possible state transitions within a model; **B** is matrix of observation probabilities (i.e., the probability of an observation at time *t* given a particular state) and *π* is a vector of initial state probabilities. We have used lab experiments, and the clinical team’s expertise to initialise the values of **T** and *π*. Note that applied techniques, including the Baum-Welch algorithm [33] and the Viterbi algorithm [33], can also be used to train the model and find the most probable series of hidden states; however, in this work we have used the data provided by our clinical monitoring team. For this purpose, the clinical monitoring team generated a set of data that was representative of probable AIA and normal state of the participant based on the observations. In conjunction to this, we used lab experiments to generate seed data for our training sets. Given *S*_*G*_2__ and *S*_*G*_3__, the participant-specific and multi-occupancy HMM models learn if an individual’s state is AIA or not and subsequently generate an output score *D*_*pf*_ and *D*_*mo*_, indicating the state of the participant. Subsequently, these score are forwarded to the decision fusion layer for final inference.

#### Decision fusion

The objective of the decision fusion layer is to optimally fuse the decision scores *D*_*CS*_, *D*_*pf*_ and *D*_*mo*_, such that the final decision is less sensitive to bias and variance. To achieve this, we define a reliability score Rs for each group. To compute Rs, the system should have the prior knowledge about the precision of individual groups for predicting class labels. A class-specific confidence estimate for each group can be computed as follows:

$$\begin{array}{c} p_{i}=\frac{TP_{i}}{TP_{i}+FP_{i}},i=1,\ldots ,M \end{array}$$ (6)

where **p**_*i*_ is the class-specific precision of the group, M is the number of class labels (i.e., which in our case is AIA or normal) and *TP* and *FP* are true and false positive rates, respectively. The reliability scores Rs for each group is computed as:

$$\begin{array}{c} Rs_{i}=\frac{p_{i}}{\sum_{i=1}^{M}p_{i}},i=1,\ldots ,M \end{array}$$ (7)

where $Rs_{i}$ for *G*_1_, *G*_2_ and *G*_3_ is learned during the cross-validation phase. The cross-validation is a common approach in machine learning for model optimisation and performance estimation. The idea is to randomly partition the data into *k*-folds, and use the *k* − 1 fold as training data and the rest as validation data for testing the model. The cross-validation process is then repeated *k* times (the folds), with each of the *k* subsamples used exactly once as the validation data. Performance scores are averaged over *k*-folds and are used as a criterion for model selection. In our experiments, we chose a standard value of *k* equal to 10 for cross-validation. The decision score $P_{j}$ output by the respective group is then multiplied with $Rs_{ij}$ to weigh it accordingly and subsequently summed to generate final scores for each class, as given by:

$$\begin{array}{c} d\left(x\right)=\arg\max_{i\in 1..M}\sum_{j=1}^{g}Rs_{ij}P_{j} \end{array}$$ (8)

where *g* is the number of groups. Finally, the class label with the highest score is reported as the label of the test event. Performance of the proposed algorithms are evaluated using the data collected from the participants in the TIHM project, which is discussed below.

## Results and discussion

In this section, we discuss our empirical evaluations of macro and micro assessment approaches.

### Macro level assessment

**Fig 4:**
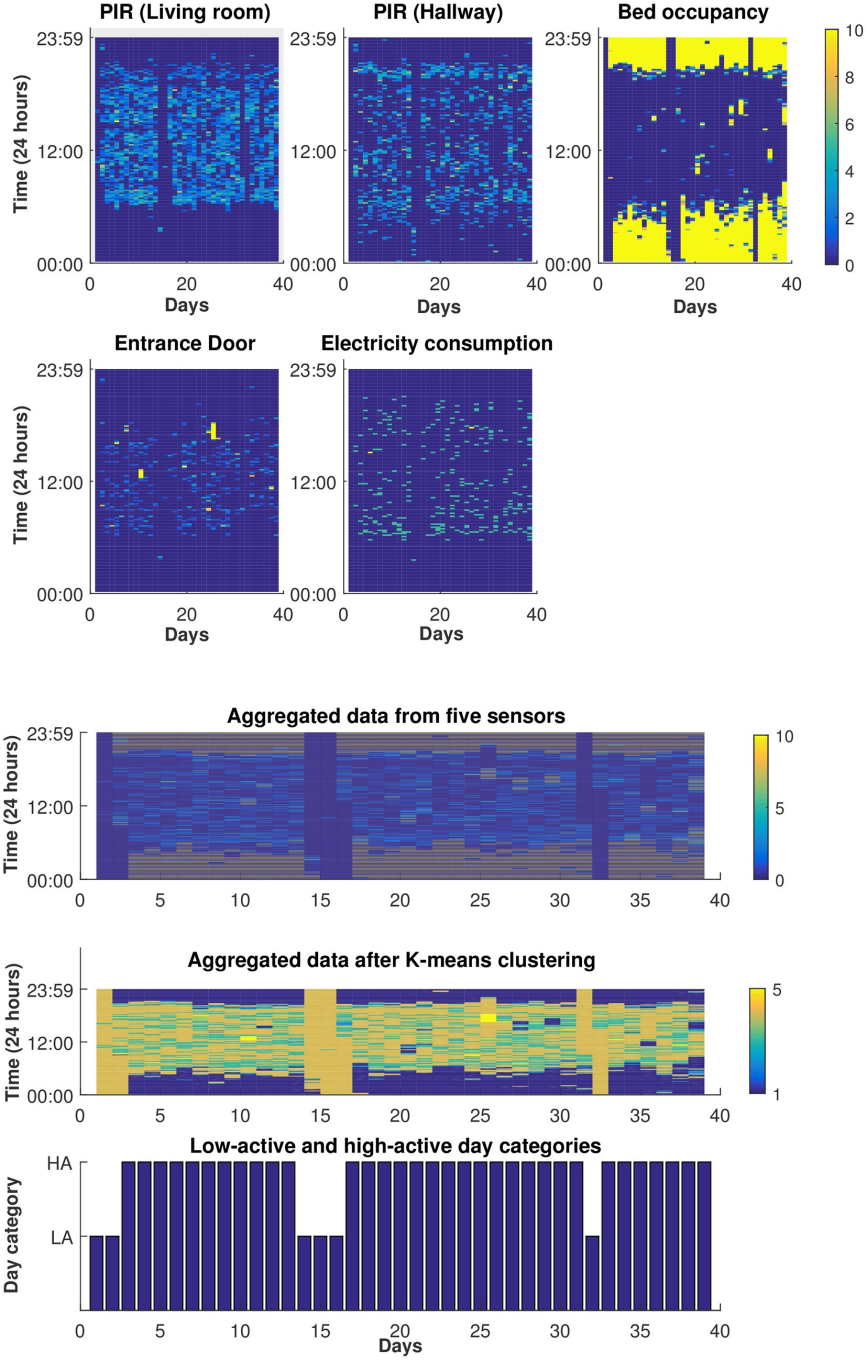
Illustration of the training data. (a) Demonstration of data collected from an individual’s home over 39 training days. The data is aggregated in 10 minutes intervals and normalised to ensure that the activity level of each sensor is ranged between 0 to 10. The value 0 in the rightmost colour legend corresponding to a dark blue colour indicates no activity and value 10 corresponding to a yellow colour indicates high activity. (b) Aggregated data from 5 sensors; where each 10 minute interval is a five-dimensional array (top). Clustering the aggregated data to map each five-dimensional window to a single state (middle). Dividing the training data into low-active (LA) and high-active (HA) categories (below).

The first algorithm (i.e., macro level assessment) is used to learn an individual’s daily patterns and detect anomalies We selected 12 participants for whom we collected data for more than three months. As an illustrative example, see Fig 4 depicting five subplots (each representing one sensory data) in which x-axis and y-axis represent day and time, respectively. The data from these sensors are then aggregated and has been represented as a five-dimensional array, see Fig 4b-top. In the next step, K-means algorithm is used to map each five-dimensional window to a single state as shown in Fig 4b-middle. In addition, a second K-means algorithm is used to divide the days into low-activity and high-activity categories, see Fig 4b-bottom. The data used in this algorithm is unlabelled and collected from uncontrolled environments where anomalies could be related to various conditions. For instance, an anomaly may originate from a technical issue (e.g. connectivity problems or sensor malfunctioning resulting in data loss), temporary changes in the recording environment (e.g. being away for multiple days or having visitors) and/or activity changes associated with a patient’s cognitive decline (e.g. being agitated and restless more often); this makes it challenging to specify the source of a detected anomaly. Therefore, in order to validate the detected anomalies, we take advantage of the complementary information that is available for each participant. In the TIHM project, we analyse the collected environmental and physiological data in (near) real-time and generate ‘environmental’ or ‘clinical’ notifications, respectively. In addition, we carry out continuous technical monitoring (e.g. for connectivity status and battery level) and generate ‘technical’ notifications. A group of healthcare practitioners (i.e. the monitoring team) monitor this information 24/7, contact participants and/or their caregivers, and confirm whether the generated notifications were reported correctly. In addition, the monitoring team keep a record of details obtained from participants (e.g. dates of hospital admission, travelling and/or family visits). This combination is used as complementary information to validate the detected anomalies.

For the overall macro level detection, we have performed weekly analysis of the target participants in order to calculate the overall sensitivity *ρ* using Eq (9).

$$\begin{array}{c} \rho =\frac{W_{val}\;\left(\#\text{Validated labelled weeks}\right)}{W_{tot}\;\left(\#\text{Total labelled weeks}\right)}=\%74 \end{array}$$ (9)

The *W*_*val*_ is the total numbers of weeks correctly labelled as anomaly whereas *W*_*tot*_ represents the total number of weeks. We have also calculated the correlation *corr* between the total number of anomalies *N*^*a*^ and the total number of validated notifications *N*^*n*^ for all the subjects using Eq (10).

$$\begin{array}{c} corr=\frac{\sum_{i=1}^{n}\left(N_{i}^{a}-\bar{N^{a}}\right)\left(N_{i}^{n}-\bar{N^{n}}\right)}{\sqrt{\sum_{i=1}^{n}{\left(N_{i}^{a}-\bar{N^{a}}\right)}^{2}}\sqrt{\sum_{i=1}^{n}{\left(N_{i}^{n}-\bar{N^{n}}\right)}^{2}}}=\%87 \end{array}$$ (10)

**Fig 5:**
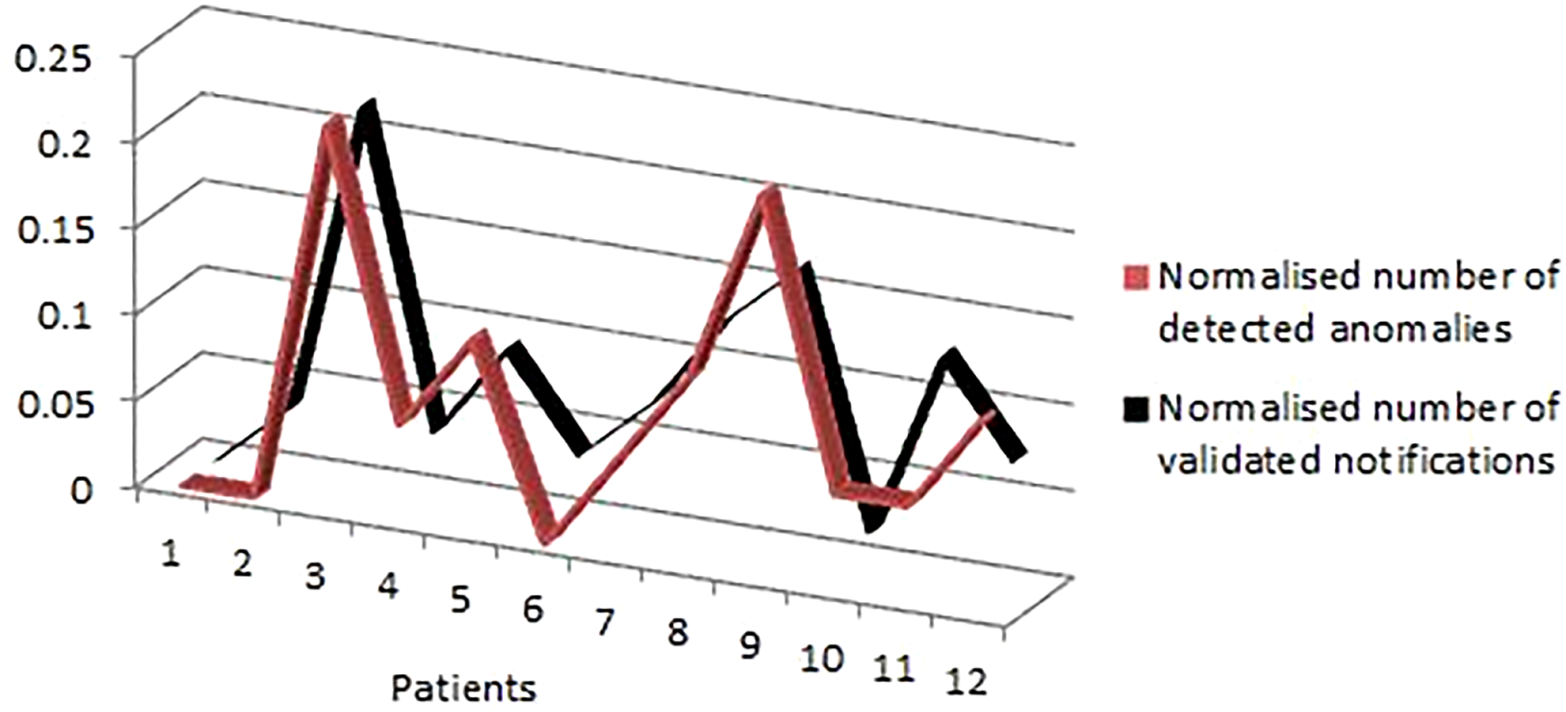
Illustration of correlation between the total number of detected anomalies and total number of validated notifications for 12 participants by the clinical monitoring team.

These notifications are generated based on various observations and measurements that are processed by the data analytics engine as discussed in detail in the TIHM project [19], and are presented to the clinical monitoring team via a secure web portal. Since these numbers originate from various sources of information, they were normalised to highlight the existing relationship. As shown in Fig 5, the high correlation between these series demonstrates that most of the study participants with a higher number of unusual patterns had more notifications in our automated monitoring system. This information can be used to prioritise patients and provide more effective and timely support at the earliest point of need to prevent escalating the conditions.

The anomalies detected in this section originate from various scenarios, for instance, technical issues (e.g. connectivity problems or sensor malfunctioning), temporary changes in the recording environment (e.g. being away for multiple days or having visitors) and/or activity pattern changes (e.g. being agitated and restless more often). The integration of the insights generated by the algorithms provide actionable information which allows us to monitor and track the general condition of people with dementia participating in this study. However, only the last case (i.e. anomalies due to symptoms such as AIA) is directly associated with individuals’ condition and/or their cognitive decline. Therefore, in the next section, we provide results of the AIA algorithm which are related to the patients’ health and well-being.

### Micro assessment

One of the many technical challenges of the TIHM project was to acquire ground truth information that is necessary to train, tune and evaluate the machine learning algorithms. Specifically, the annotation of AIA symptom is rather challenging, since it is susceptible to human bias. To approach the problem of detecting possible AIA events in an automated fashion, we adopted a statistical threshold based approach, namely median absolute deviation (MAD) [34] detection to detect the presence of outlying values. This approach makes use of the training data to compute the MAD value that acts as a threshold to generate alerts for detecting the change in patient’s observation and measurement data. Once the alerts were generated, the clinical team at the hospitals obtained the true annotation of these events by contacting the caregiver and verifying the alerts. Subsequently, the labels of these initial events were duly updated in the database. In our empirical evaluation, the algorithms generated a total of 74 AIA event flags that were duly verified by the clinical team as AIA or non-AIA and were used in the cross-validation phase for the performance estimation of our models.

**Fig 6:**
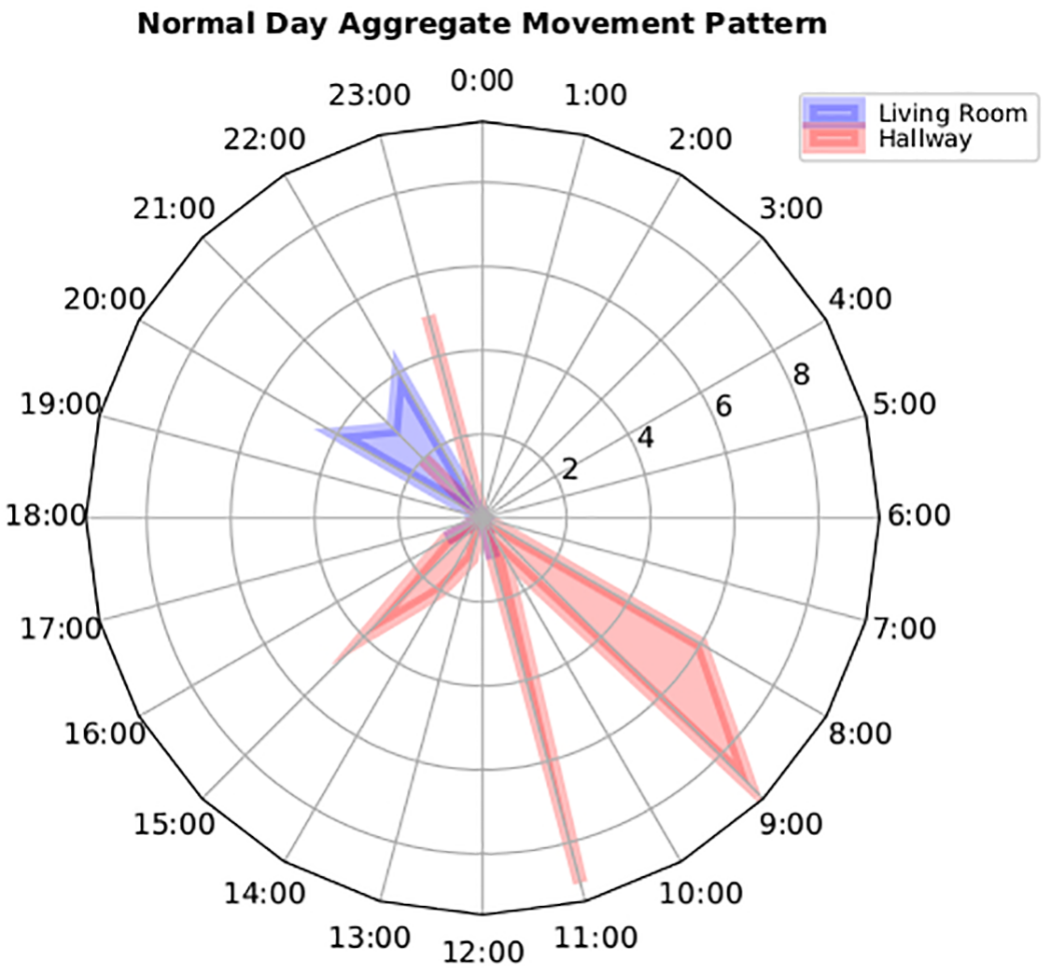
A normal movement pattern reported in the living and hallway for Patient #12.

**Fig 7:**
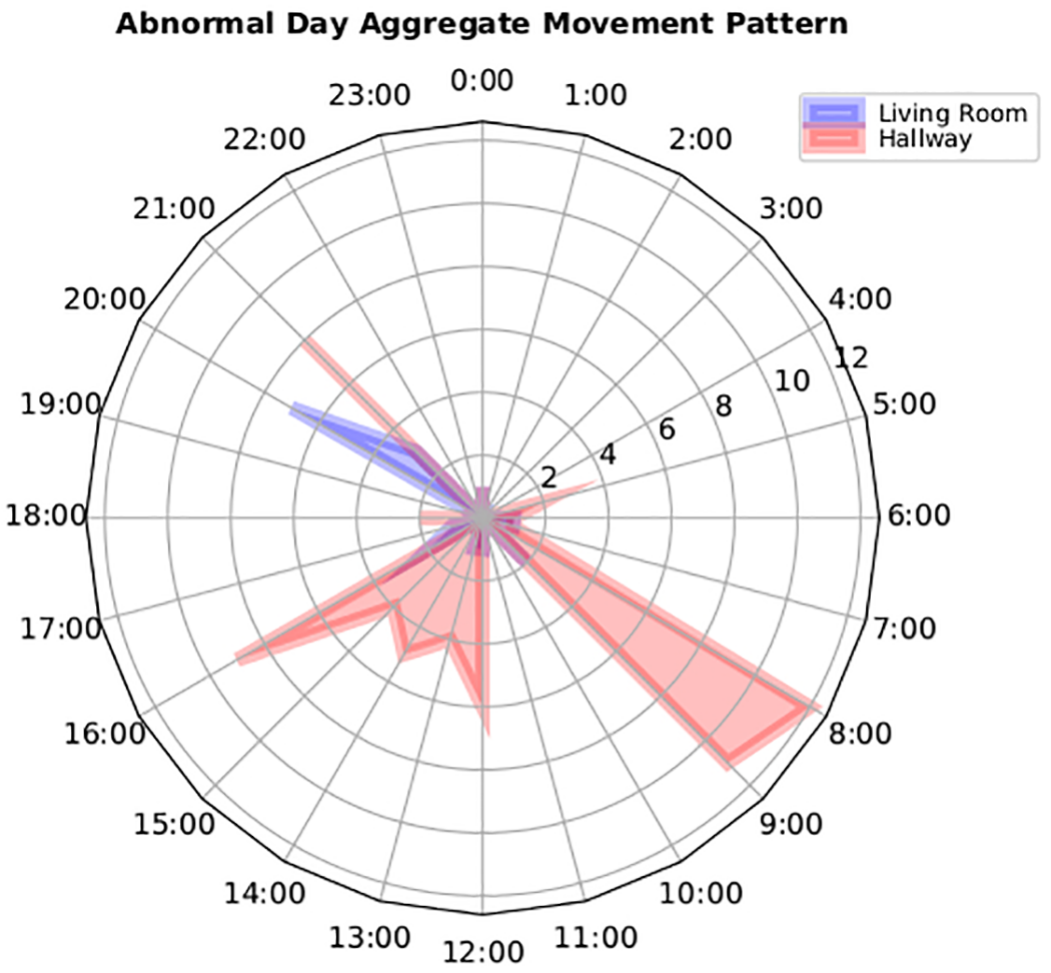
Abnormal movement pattern reported in the living and hallway for patient #12 due to presence of guests.

Although, the naive approach helps provide a clue to the system with regards to abnormality, however in the context of AIA detection, simple statistical thresholds generates a large number of false positives. Our naive approach takes into account the re-occurring movement patterns of the user based on the time of the day and the reported motion sensor values, enabling it to classify normal and abnormal movement patterns. For example, movement pattern of participant A as shown in Fig 6 is flagged as normal by the algorithm based on its probability of re-occurrence. Fig 6 exhibits that participant is normally active in the hallway during early morning between 8:00 AM to 11:00 AM. Likewise, we see a clear movement pattern in the living room between 20:00 to 22:00 PM. In contrast, an abnormal movement pattern would differ from the normal pattern as shown in Fig 7. This figure shows that not only there is an overall increased movement activity reported in comparison to normal day pattern but also the successive movement patterns between the hallways and living room during the midday indicates an unusual routine. Upon contacting the patient, our clinical team found that the abnormality in the movement is recorded due to the presence of guests in the house. This explains that statistical thresholds relying on activity count alone is not a reliable metric to generate alerts. Instead, the context of the movement pattern and the relationship between different activities must be taken into account to accurately detect AIA events. To address this issue and reduce the number of false positives, we decoupled the participant-specific movement model and the aggregated movement model using a weighting algorithm described in the methodology section.

**Fig 8:**
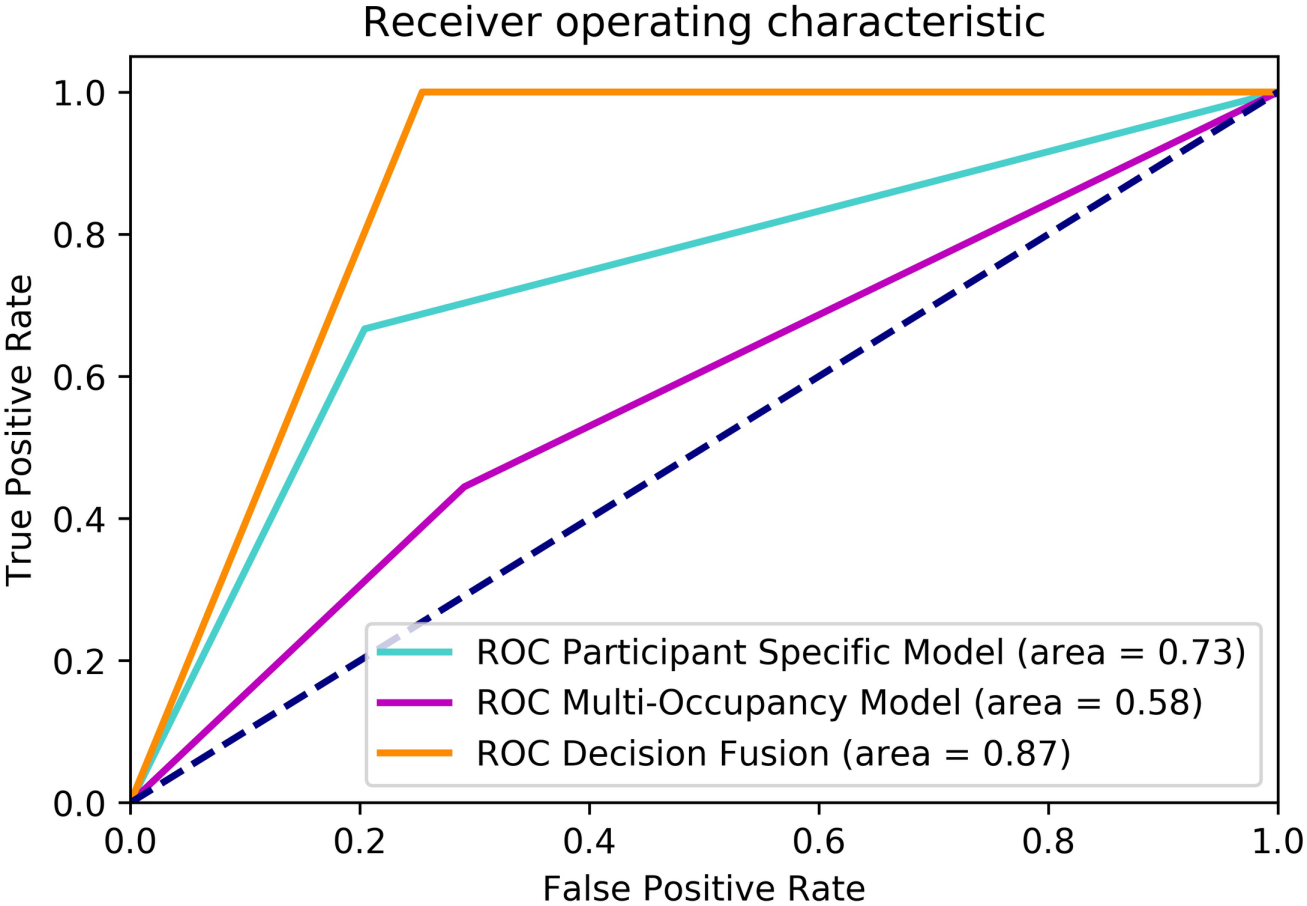
Receiver operating characteristic curves for multi-occupancy, participant-specific and decision fusion models for the detection of AIA.

A standard evaluation method, namely Area Under the Curve (AUC) measure associated with receiver operating characteristic (ROC) curve [108] is adopted to quantify the performance of our algorithms. The ROC curve is a plot of fraction of true positives versus false positives varied at various threshold settings. The AUC is the area under the curve which summarises the ROC curve in one number. AUC score of 1 means that the model/classifier has made no classification errors, whereas a random classifier will likely to achieve an AUC score of 0.5. We report the model performance estimate in terms of AUC score averaged over 10 folds. The total number of AIA flags generated by the system is used as a test data and the performance of the participant-specific, multi-occupancy and decision fusion models is measured against the duly validated labels provided by the clinical teams. It can be clearly seen from Fig 8 that multi-occupancy model alone achieves a low AUC score, indicating lots of miss-classification of AIA events. Although, we inferred that multi-occupancy patterns do correlate with the AIA symptoms to an extent; but is not reliable enough to be used as a final inference alone. Using only this information we achieved an AUC score of 58%, as shown in Fig 8. On the other hand, the participant specific model has shown a performance improvement over multi-occupancy model and reports a AUC score of 73%. However, the reported evaluation results discussed above and in Fig 8 clearly suggest that decision fusion outperforms the previous two approaches and it considerably improves the reliability of the system in detecting AIA. The proposed decision fusion strategy yields a significant improvement of 14% and 29% against the participant-specific and multi-occupancy models, respectively. As reported in Table 1, the weighted precision and recall metrics of the fusion model are above 80%, which depicts that decision fusion helps improves the overall precision and reliability of AIA event classification.

**Table 1:** Classification insights into decision fusion model.

Decision Fusion Model	Precision	Recall	f1-score	Max Precision	Max Recall
Non-AIA Class	0.85	0.89	0.84	1	1
AIA Class	0.72	0.70	0.70	1	1
Weighted Average	0.81	0.83	0.80	-	-

## Conclusions and future work

The Technology Integrated Health Management (TIHM) project utilises IoT-enabled technologies to monitor people with dementia in their living environments. One of the key contributions of TIHM is developing machine learning and data analytics algorithms that combine environmental and physiological data to learn and discover changes in participant’s health and well-being. This paper presents a novel approach to profile the daily patterns of people with dementia. The proposed algorithms provide actionable information to assist the diagnosis and decision making of clinicians, while being non-invasive and privacy-aware. In this paper, we target the challenge of learning the daily activity patterns of people who are affected by cognition decline in order to detect changes in their routines. In conjunction, by analysing the continuous data stream from sensory data sources, we infer Agitation, Irritation and Aggression (AIA) events. Our proposed hierarchical fusion algorithm shows that a data-driven detection of abnormal patterns such as AIA is feasible by analysing the raw observation and measurement from different sensory devices. Our model learns the individuals’ repetitive patterns and performs (near-) real-time activity classification, segmentation and abnormality detection. We have demonstrated the efficiency of our proposed algorithms by conducting classification and prediction-based evaluations. The future work will focus on validation of the proposed methods on larger population sizes in a longer time-span. We also plan to develop predictive and incremental learning models to provide more information and insights that can be extracted from monitoring physiological and environmental data. This work has been a step towards using sensory and automated observation and measurement data in real clinical settings. We showed how environmental and sensory data is used to improve the clinical decision making and enhance the care and support given to patients and their caregivers.

## Supporting information

S1 Algorithm Anomaly detection algorithm. (PDF)

S2 Algorithm Decision scoring by the expert system. (PDF)

S3 Algorithm Label sequence generation by categorisation block. (PDF)
