# Supplementary material for: Health management and pattern analysis of daily living activities of people with dementia using in-home sensors and machine learning techniques
Source: PLoS One. 2018 May 3;13(5):e0195605. doi: 10.1371/journal.pone.0195605 (PMC5933790; doi:10.1371/journal.pone.0195605)
Supplement: S1 Algorithm — (PDF) [file pone.0195605.s001.pdf]

Supporting information

S1 Algorithm: Anomaly detection algorithm

1. Filter and pre-process the original data. This includes defining a common sampling rate for the given environmental sensors (e.g. one sample per minute)
2. Aggregate the integrated data within ten minutes intervals (each day is represented via $144(24 \times 6)$ windows and each window is an array of n observations)
3. Cluster the daily data into categories; In this case to $D1$ and $D2$ (low-active and high-active days)
4. Cluster individual windows in order to map each window into a single state, i.e. the data is distilled into a discrete format: at time interval τ_t the user is in state s_i.
5. Separate the historical data into training, verification and test datasets. In this experiment we have used training (%50), verification (%20), and test sets (%30)
6. Construct the Markov chain model for each cluster and learn the corresponding training transition matrices
7. Calculate the entropy rate ξ_T via Eq. (4)
8. Repeat steps 3-4 for the verification set and calculate the entropy rate for each day in this dataset
9. Determine a confidence interval (expected variation) for entropy rate values, see Eq. (5)
10. Repeat steps 3-4 for the test data, calculate the entropy rate, and compare with the confidence interval to detect anomalies
