# Supplementary material for: Health management and pattern analysis of daily living activities of people with dementia using in-home sensors and machine learning techniques
Source: PLoS One. 2018 May 3;13(5):e0195605. doi: 10.1371/journal.pone.0195605 (PMC5933790; doi:10.1371/journal.pone.0195605)
Supplement: S2 Algorithm — (PDF) [file pone.0195605.s002.pdf]

Supporting information

S2 Algorithm: Decision Scoring by the Expert System

Input: Aggregated hourly measurements O_i reported by CS_i, Clinical Thresholds T_i^H and T_i^L for respective $CS_i \quad \forall i \in G_1$

Output: Decision score D_{CS}

Data: Fetch Clinical thresholds T_i^H and T_i^L from the database, where H and L indicate higher and lower thresholds values

Initialise D_{CS} to 0

foreach CS_i **do**

Compare the O_i against the thresholds T_i^H and T_i^L

if O_i lies outside the range **then** Increment D_{CS} value

else Do not change the D_{CS} value

end
