# Supplementary material for: Health management and pattern analysis of daily living activities of people with dementia using in-home sensors and machine learning techniques
Source: PLoS One. 2018 May 3;13(5):e0195605. doi: 10.1371/journal.pone.0195605 (PMC5933790; doi:10.1371/journal.pone.0195605)
Supplement: S3 Algorithm — (PDF) [file pone.0195605.s003.pdf]

Supporting information

S3 Algorithm: Label Sequence Generation by Categorisation Block

Input: Aggregated hourly measurements P_x from PIR Sensors PS_x where $x \in G$

Output: A sequence of alphabetical labels S where $|S| = |G|$

Data: Fetch pre-computed Upper Boundary B_x^u and Lower Boundary B_x^l values of the adjusted box-plot from the database against each sensor in G

Initialise S to be an array of empty string

foreach PS_x **Initialise**

Compare the P_x against the thresholds B_x^u and B_x^l

Identify the corresponding label set A_x for mapping

if P_x lies outside the normal range **then** select a label L that represent abnormality where $L \in A_x$

Select a label L that represents normality where $L \in A_i$

Append the Output to S

end
